# Analysis of the Role of Female Hormones During Infection by COVID-19

**DOI:** 10.1055/s-0041-1740208

**Published:** 2021-12-21

**Authors:** David Balbino Pascoal, Isabela Macêdo de Araujo, Lorenna Peixoto Lopes, Cristiane Monteiro da Cruz

**Affiliations:** 1 Department of Medicine, Centro Universitário Cesmac, Maceió, AL, Brazil; 2 Department of Gynecology and Obstetrics, Universidade Federal de Alagoas, Maceió, AL, Brazil; 3 Department of Medicine, UNIT University Center, Maceió, AL, Brazil

**Keywords:** woman, hormones, coronavirus, thrombosis, contraception, mulher, hormônios, coronavírus, trombose, contracepção

## Abstract

Women have metabolic, immunological, and genetic variables that ensure more protection from coronavirus infection. However, the indication of treatment for several pathologies and contraception is determined by hormones that have adverse effects and raise doubts about their use during the COVID-19 pandemic. Therefore, the present study searches women specificities and the relation between female sexual hormones and COVID-19, and reports the main recommendations in this background. To this end, a review of the literature was conducted in the main databases, auxiliary data sources, and official websites. Therefore, considering the hypercoagulability status of COVID-19, the debate about the use of contraceptives due to the relative risk of thromboembolic effects that they impose arises. However, the current available evidence, as well as the recommendations of main health organs around the world, demonstrate that the use of hormonal contraceptives must be maintained during the pandemic.

## Resumo

As mulheres possuem variáveis metabólicas, imunológicas e genéticas que conferem maior proteção à infecção pelo coronavírus. Todavia, a indicação de tratamento para certas patologias e para a contracepção é determinada por hormônios que possuem efeitos adversos e levantam dúvidas quanto ao seu uso durante a pandemia da COVID-19. Desta forma, o presente estudo busca investigar as especificidades da mulher e a relação dos hormônios sexuais femininos com a COVID-19, assim como relatar as principais recomendações neste contexto. Para isso, realizou-se uma revisão da literatura nas principais bases de dados, fontes auxiliares de dados e sites oficiais. Portanto, considerando o estado hipercoagulável da COVID-19, surge o debate quanto à utilização de contraceptivos pelo seu risco relativo de efeitos tromboembólicos. No entanto, as atuais evidências disponíveis, assim como as recomendações dos principais órgãos de saúde do mundo, demonstraram que o uso de contraceptivos hormonais deve ser mantido durante a pandemia.

## Introduction

Coronavirus is a disease with high transmissibility and mortality, with 15,785,641 confirmed cases around the world accumulated until July and, in Brazil, a total of 2,343,366 cases according to the World Health Organization (WHO). 1 The infection defies science to find therapeutic alternatives to its containment and cure. It is known that some specific conditions are analyzed as determining factors to the high morbimortality of patients. Among these, epidemiological data demonstrated that women show less cases of infection by the virus and a lower mortality rate. 2

Thereby, some of the factors that can influence in the differences between genders are determined by metabolic, genetic, and immunological variables, and studies demonstrate that these mechanisms provide more protection to women. 3 However, the main features in the treatment of pathologies or in contraception must be critically analyzed when facing the pandemic. After all, with social isolation, health services have their attendance adapted to a reality of social isolation, which does not allow integral monitoring to the needs of women. 4 In addition, the indication of treatment for some pathologies and contraception are determined by hormones that have side effects and raise doubts about the right use when facing COVID-19. 5 Consequently, the present study aims at investigating the main variables of females, such as the relation between female sexual hormones and COVID-19, as well as at reporting alternatives for the rational use of contraceptives in the pandemic background.

## Methods

The present study consisted of a literature review that performed searches in the Medline (by PubMed), Biblioteca Virtual em Saúde (BVS, in the Portuguese acronym), Google Scholar, ScienceDirect, and Cochrane Library and Clinical Trials databases. In addition, other auxiliaries and official Web sites were consulted: Opengray, Agência Nacional de Vigilância Sanitária (ANVISA, in the Portuguese acronym), Federação Brasileira das Associações de Ginecologia e Obstetrícia (FEBRASGO, in the Portuguese acronym), Fédération Internationale de Gynécologie et d'Obstétrique (FIGO) and WHO. The studies were tracked, systematically, according to the strategy of population, intervention, comparison, and outcomes (PICO), by the use of the MESH descriptors *women* , *girl* , *contraceptive* , *hormone replacement* , *estrogen* , *progesterone* , *COVID* - *19* , *coronavirus* , *SARS-CoV-2* , *thrombosis* and *coagulation* . The present study was performed during the month of July and included articles published since 2016. Due the heterogeneity of the studies, there was no quantitative analysis of the results.

### Biological Differences between Genders

Women are less susceptible to SARS-CoV-2 infection and have lower morbimortality rates, which are determined by hormonal and chromosomal specificities that ensure increased protection to them. Therefore, with the worldwide dissemination of the disease, epidemiologic data showed that males are more affected and have increased chances of developing the severe stage of the disease. 5 It is known that behavioral factors, such as precarious health care and smoking, as well as the presence of comorbid conditions, influence in the differences between genders and, consequently, in the severity of the disease. Furthermore, it was observed that biological features, including chromosomes and sexual hormones, contribute to the increased risk of poor prognosis of the patient. 3 Therefore, the genetic aspects linked to the X chromosome and the female steroids hormones activity positively modulate both innate and adaptive immunity, ensuring a more efficient, stronger, and extended response to women. 6 7 8 It is believed that this advantage of females is due to the influence of estrogen levels and to the presence of two polymorphic X chromosomes. 6 9

### Genetic and Molecular Aspects

The genetic differences between genders, given by the extra X chromosome, ensures an increased variety of proteins and cellular population to females. 6 In the COVID-19 background, it is known that the angiotensin 2 converting enzyme ( *ACE2* ) gene is located in the X chromosome, exerting a significant role on the maintenance of arterial pressure and regulation of fluids, which reduces the risk of possible pulmonary damages. 3 10 Besides that, genes coded by this chromosome also positively regulate the immunological function by rising TCD4 cells, antibodies, and Toll-like receptors (TLR3, TLR4 e TLR7). 10 The TLR7 has the capacity of escaping from the inactivation of the X chromosome, increasing its expression in female immune cells. 3 Therefore, it is able to detect simple tape RNA, proper of the infection by SARS-CoV-2 and, then, reduce viral titers. 10 It is also known that the *FOXP3* gene is present in the X chromosome, modulating the function of regulating T-cells (Treg). 11

### Hormonal Aspects

Hormones are chemical messengers that interact with receptors from different cells, leading to biochemical reactions that lead to specific biological responses. 12 Therefore, 17β-estradiol exerts a reducing effect on the expression of *ACE2* in the lungs, regardless of the chromosomal complement. 10 Furthermore, the protection of women is due to the connection to its receptor, inducing the production and transduction of signs of cytokines. 9 13 Estrogen levels influence the type of immune response, since low doses activate Th1 response with the release of proinflammatory cytokines (interleukin-1 [IL-1], IL-6, and TNF) – while higher doses result in their suppression, increasing Th2 response and humoral immunity. Besides, this hormone induces the expression of TLR4 on the surface of macrophages. Concomitantly, progesterone has anti-inflammatory effects, with the transformation of Th1 response to Th2, along with the increase of the release of IL-4, IL-5 and IL-10. In addition, it increases the number of Treg cells and decreases levels of circulating Th17, and it may also antagonize TLR and NF-κB pathways. 14

In the study by Suba, 5 it was demonstrated that female mice are less susceptible to SARS-CoV infection. Male mice presented a viral titer considerably higher, in addition to intense inflammatory response, adding mortality risk to this gender. It is worth pointing out that female mice infected by the respiratory virus and submitted to ovariectomy or to treatment with estrogen receptors (ERs) antagonists had their morbimortality rate increased, which shows the importance of the role of estradiol on the activation of ERs for protection against the virus.

The impacts of estrogen on the regulation of the immune system depends on the concentration of the hormone, as well as on its distribution, its density, and on different types of ERs present in immunological cells. It is known that women's immune responses can vary with different concentrations of estrogen during the menstrual cycle and that the capacity of their immunological response is substantially reduced when they reach menopause, since estrogen is now replaced by estrone, which presents lower action capability. 10 14 Therefore, menstrual regularity represents a major impact for the protection against COVID-19. 9

### Regulation of Immunological Response

Females present a higher quantity of immunoglobulin G (IgG) antibodies in the beginning of the infection by SARS-CoV-2, as well as women who present with the severe type of the disease. Hence, it is observed that an increased percentage of IgG in female patients provides more protection against the evolution to the severe type of the disease or to death. 15

Estrogen positively acts on the superior and inferior airways. In the nasal cavity, it has the capability of optimizing the local innate immune response, besides stimulating the reactivity of the mucosa, increasing the production of antiviral substances, such as electrolytes, mucins, lactoferrin, oligosaccharides, immunoglobulins A and G, and hyaluronic acid. The latter is also stimulated by hormone in the oral cavity, ensuring better oral hydration. Besides that, the increase in the production of antiviral agents also happens in the lungs, being sustained by the effect of progesterone, which helps in the pulmonary repair by the release of amphiregulin. 16

Estrogen receptors are classified as types α or β, which are expressed in the immune system cells in different ways, since ERα is highly expressed in T-lymphocytes and ERβ in B-cells. 7 14 It is also known that ERs are expressed in innate immune cells, such as neutrophils, macrophages, and monocytes. Thus, estrogen-activated ERs regulate the development of immune cells, positively modulating the innate and adaptative responses. 5

From the activation of ERs in innate cells, there is the release of proinflammatory cytokines (IL-12, tumor necrosis factor-α [TNFα]) and of a chemokine (CCL2). 17 These are responsible for stimulating the expression of aromatase that promotes the conversion of androgen into estrogens, which potentiates the female immune response. The activation of ERα in T-cells promotes the release of interferon types I and III (IFN), an essential factor for the inhibition of viral replication. 5 9 Dendritic cells are also influenced by estradiol signaling via ERα, which are responsible for the increase of the number of cells during inflammation. 9

The main dendritic cells involved in antiviral response are plasmocytoids (pDCs), which are responsible for producing more IFN I when compared with males. 5 9 The pDCs, when stimulated by TLR7, present higher levels of IFN 5 regulator factor (IRF-5) and higher production of IFNα, which is important against the virus. 3 14 It is worth noting that an experiment with female mice demonstrated that the regulation of the transcription of IRF5 is controlled by the signaling of ERα. 14

**Fig.1:**
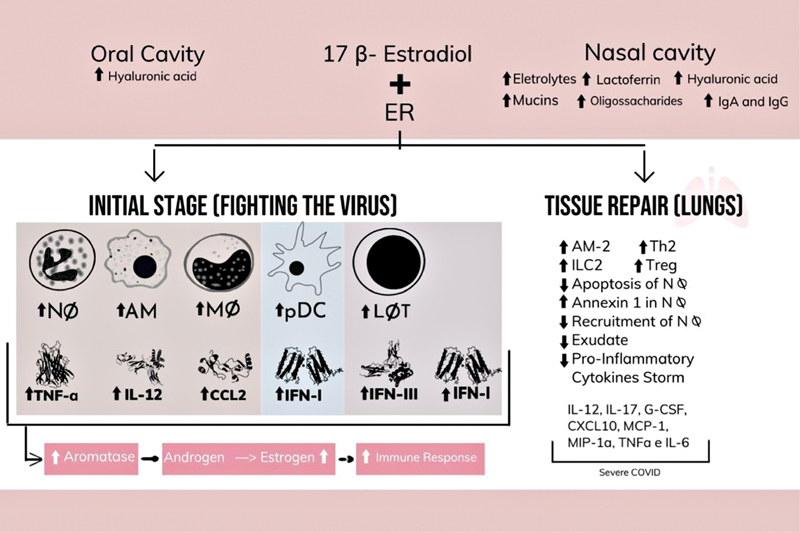
Immunological mechanisms induced by estrogen.

At the end of the viral infection, the activation of ERα by high levels of estradiol stimulates a type 2 response of innate immune cells (ILC2), myeloid cells, and alveolar macrophages (AMs). 7 These, in turn, have the ability of producing antiviral mediators, IFN I chemokines that recruit monocytes to the lungs. 5 16 Thus, estrogen reduces the release of proinflammatory cytokines in monocytes and macrophages, improves the expression of annexin-1 of neutrophils without increasing their activation, in addition to delaying apoptosis of neutrophils, attenuating viral hyperinflation and favoring tissue repair. 9 Fig. 1 describes the immunological mechanism regulated by estrogen in the face of the infection by respiratory virus, as well as the process of restoration of the lungs.

In a study conducted by Channappanavar et al., 8 it was observed that 72 hours after SARS-CoV infection, the levels of chemokines (CCL-2 and CXCL-1) and of proinflammatory cytokines (IL-6) remained the same or even increased in the lungs of male mice, when compared with females. This experiment was reinforced by Suba, 5 who demonstrated that high concentrations of estrogen in female mice, after the same period of the previous study, reduced the cytokines storm caused by SARS-CoV, while the high levels of inflammatory in male mice lasted longer.

Therefore, the effects of female sex hormones on the modulation of molecular, biochemical, and immunological mechanisms in viral infection are evident. Nevertheless, two research are in progress at ClinicalTrials and, based on these effects, they seek therapeutic alternatives to COVID-19 through estrogen and progesterone. 18 19

**Fig. 2:**
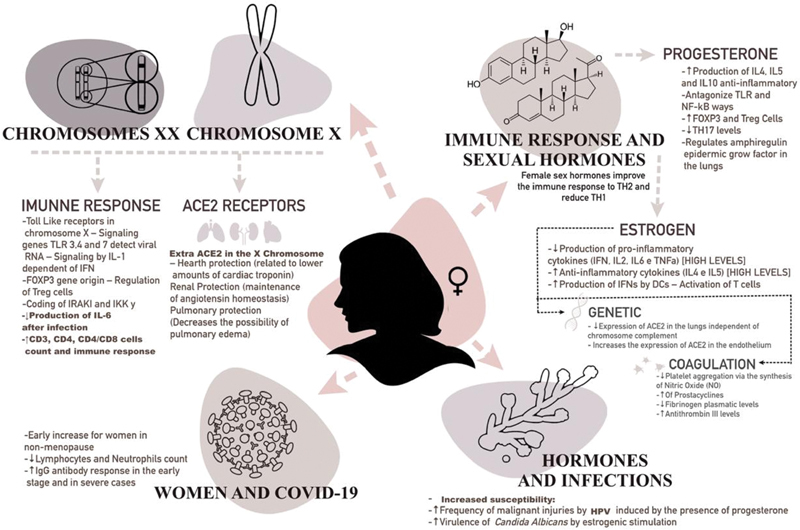
Biological differences intrinsic to the female gender.

Given the evidence described, the genetic, molecular, hormonal, and immunological differences of women are perceptible, providing, therefore some advantage to females in the face of various infections. These differential characteristics are described in Fig. 2 .

## COVID-19 and Thromboembolic Events

Within pathophysiology, the SARS-CoV-2 virus is responsible for damaging effects that, after the binding of the virus to the receptors in lung pneumocytes, can reach other organs of the human body due to its affinity with ACE2 receptors ECA2. 20 Therefore, besides causing a decline on respiratory function, COVID-19 causes systemic effects that worsen the morbimortality of infected patients. 21

The main systemic complications are based in coagulation abnormalities suggestive of hematological pathologies such as thrombotic microangiopathy or disseminated intravascular coagulation (DIC) in severe patients. In this context, venous thromboembolism (VTE) and arterial thromboembolism (ATE) have been frequently observed in intensive care units (ICUs). 2 According to Klok et al., 22 40% of ICU patients with COVID-19 presented thromboembolic complications.

The vascular endothelium presents lesions due to cytopathic damage caused by the virus and to inflammation caused by its generation of an endothelial dysfunction. 23 In addition, lesions in endothelial cells result in the release of plasminogen activators, which are the explanation for a high concentration of D-dimer and fibrin in severe patients with COVID-19. 2 23 In the study by Guan et al., 24 46% of critical ill patients showed higher levels of D-dimer.

Autopsies of these patients revealed fundamental anatomopathological aspects for the elucidation of the characteristics of viral activity. The detection of microthrombi in the lumen of pulmonary vessels could be better elucidated by immunohistochemical analysis, which revealed a high platelet production per CD61+ megakaryocytes. 25 In the study, it was found that the increase in the number of platelets was due to the activation of the complement system observed by the deposition of complement system protein subtypes in the microvasculature, 26 explaining the relationship between coagulopathy and immune response to COVID19. 27

Hypercoagulability is determined, mainly, by the increase of prothrombotic factors in critically ill patients. 23 Together, the increase in the concentration of proinflammatory cytokines such as TNF-a and of interleukins such as IL-1, IL-6, and IL-10 may induce the expression of tissue factor in mononuclear cells and, consequently, activate the coagulation chain and the generation of thrombin. 2 Furthermore, a study has shown that, in critically ill patients, an increase of platelet-monocytes aggregates – inducers of tissue factor expression – occurred, and this event was also related to an increase in markers such as fibrinogen and D-dimer. 28

Critically ill patients with COVID-19 are hospitalized and, therefore, have movement limitations that alter their hemodynamics and fit the *Virchow* triad. Therefore, the venous stasis determined by the immobilization of these patients is strongly aggravating for the genesis of thrombotic events. 23 Moreover, special situations with potential dysfunctions in coagulation should be considered; after all, pregnant women, for example, have a physiological state of hypercoagulability. 29

## Women's Health, Family Planning and COVID-19

Medical services, including those of Gynecology and Obstetrics, were strongly shaken and had to adapt to a new context with obstacles in providing care due the COVID-19 pandemic. However, it is vital that services aimed at women's health such as reproductive health and contraception be maintained. 4 According to official recommendations, these services should institute continued care, offering unrestricted access to treatment for clinical and pathological conditions and family planning. 30 31

It is noticeable that no matter how there is no evidence of worsening in pregnant patients with SARS-CoV-2 or of vertical transmission, the possible intercurrences arising from the pregnancy process that may require more advanced medical care are concerns in this panorama. After all, given the severity and the speed of the spread of the virus, almost all hospital care flows were directed to these patients. Therefore, when possible, it is recommended to postpone pregnancies. 32

Thus, contraceptives are primordial within this premise of maintaining sociodemographic levels. 4 However, according to Riley et al., 33 during the pandemic, there was a decrease of ∼ 10% in contraceptive use in 132 countries from low- and middle-income countries. This represents a shortage of contraception for 48,6 million women and, consequently, ∼ 15 million unwanted pregnancies. This can be explained by the difficulty of access to these methods, 34 but also by the hypothesis that contraceptives, due to their thromboembolic risk, 5 have a synergistic effect in the view of the thrombotic pathophysiology of COVID-19. 2 22 23

## Hormonal Therapy and Thromboembolic Risk

Evidence determines that endogenous estrogen has a protective effect regarding COVID-19. 5 9 10 However, SARS-CoV-2 has evolved beyond the understandable point of previous experiences and challenges theories that suggest vascular protection. As much as this effect has been documented, exogenous estrogen can increase the activation of coagulation factors, increasing the thrombotic risk. 35 36 In this view, oral contraceptives with estrogen are excellent drugs in terms of pregnancy prevention; however, in certain cases, they have determined unpredictable thromboembolic complications such as mesenteric vein thrombosis, 37 VTE, 38 stroke, and acute myocardium infarction. 5

As with contraception, menopausal hormone replacement therapy also needs to be evaluated, since women who use it have a 2.9 times greater risk of presenting thromboembolic events than those who do not. 39 Similarly, despite its low incidence, the risk of thromboembolism during fertilization procedures is similar that in pregnant women, which is 10 times higher than that in young women of reproductive age. 23

## Hormonal Formulations and Thrombotic Risk

The use of contraceptives is a thromboembolic risk factor for women susceptible to developing hemolytic diseases. 40 However, even though the risk of hemostatic disorders is 4 times higher in contraceptive users, the absolute risk remains low and is lower than the risk associated with pregnancy. 41 Nevertheless, at ∼ 35 years of age, the risk of thrombosis in women who use contraceptives is the same as that in pregnant women. 40

In the contraceptive formula, estrogen fits as the ethologic agent for hemostatic disfunctions. According to Gialeraki et al., 42 the risk of thrombosis is double in women who take pills containing high doses of estrogen. Regarding the type of estrogen, the formulations with estradiol (E2) are relatively better than those with ethinyl estradiol (EE). 43 Similarly, estrogens administrated vaginally in low doses were not related to an increased risk of developing thrombosis. 39

Regarding progestogens, there is a global consensus that, except for medroxyprogesterone acetate deposit, there is no significant association between the isolated use of progestin and increased VTE prevalence. Even in high doses of Levonogestrel or ulipristal – present in the formulation of emergency pills, isolated, are also not associated with vascular events. 40 44

Regarding combined oral contraceptives (COC), the risk of developing VTE is much higher if compared with other isolated formulations. This risk dependent on the dose of EE together with gestodene, desogestrel, cyproterone acetate or drospirenone (3 ^rd^ and 4 ^th^ generation progestins). All of these are beneficial but are similar in terms of VTE risk and are ∼ between 50 and 80% more likely than Levonogestrel. 40 42 45 This fact is evidenced by the studies presented in Chart 1 . 46 47 48 49 50

**Chart 1:** Cohorts of relative risks of thrombotic events in different contraceptive formulations

Authors	Contraceptive formula	Thrombotic event	Multivariate analysis (relative risk)
Laliberté et al. 50	Transdermal estradiol	VTE	0.67
	Oral estradiol	VTE	
Larivée et al. 47	Drospirenone	ATE	0.89
	Levonorgestrel	ATE	1
Weill et al. 48	Ethinyl estradiol (20 µg)	PE	0.75
		STROKE	0.82
		AMI	0.56
	Levonorgestrel	PE	1
		STROKE	1
		AMI	1
	Desogestrel	PE	2.16
		STROKE	0.96
		AMI	1.01
	Gestodene	PE	1.63
		STROKE	0.96
		AMI	0.49
	Ethinyl estradiol (20 µg) + Levonogestrel	PE	0.74
	Ethynil estradiol (20 µg) + Desogestrel	PE	0.75
	Ethynil estradiol (20 µg) + Gestondene	PE	0.94
Hugon-Rodin et al. 49	Desogestrel	VTE	1.4
	Cyproterone	VTE	1.71
	Drospirenone	VTE	1.99
Dinger et al. 46	Levonogestrel	VTE	0.5

Abbreviations: AMI, acute myocardium infarction; ATE, arterial thromboembolism; PE, pulmonary embolism; VTE, venous thromboembolism.

Chart 1 describes five cohorts 46 47 48 49 50 that evaluated the types of estrogen and progestogen – as well their combination – and the relative risk of thrombotic events within distinct multivariate analyses. The analysis of the studies mentioned in **Chart 1** may have limitations due to do not consider the dosage, do not control all factors of confusion and different study methodologies.

Therefore, in relation to thromboembolic complications, Levonogestrel and the lowest possible dose of EE are associated with a lower risk of VTE (if only the risk is taken into the account), especially in novice users. After all, thromboembolic complications attributed to the use of hormones are greater at between 6 and 12 months of initial use. 42

## Thromboembolic Physiopathology of Contraceptives

The thrombotic physiology of these formulations stems from several complex multifactorial disorders with no defined causal factor. Thrombotic events depend on congenital and acquired conditions ruled as the main risk factors among users of hormonal contraception, such as acquired conditions such as surgery, pregnancy, age, smoking etc., and congenital alterations such as gene mutations related to coagulation factors (Leiden factor V gene, prothrombin gene, FGG etc.). 45 In the cohort carried out by Dulicek et al. 51 in users of hormonal contraception who had thrombotic events, 44% of the patients that had VTE had thrombophilia and, in the group of patients who had arterial effects (CVA), 50% were smokers.

Thus, epidemiologically, the use of contraceptives is associated with increased levels of fibrinogen, prothrombin, D-dimer, plasminogen tissue activator, plasminogen, and coagulation factors VII, VIII, and X, as well as with abnormal resistance of C-reactive protein (CRP). 41 42 All of these altered markers signal to a procoagulant activity, increased fibrinolytic activity, and endothelial injury, which result in hypercoagulability that, consequently, leads to thrombotic events. 40

To explain this pathophysiology, the abnormal function of CRP can be analyzed. 52 It is perceived that women who make use of COC present resistance to CRP, whose effect may be related to the disturbance of sex hormone binding globulin (SHBG) induced by different classes of progestogens. After all, depending on the level of antiandrogenicity of the formulation, the levels of SHBG may vary and decrease the levels of inactivation of coagulation factors by CRP. 42

Another important aspect to elucidate the thrombotic pathophysiology of contraceptives is related to liver effects, 53 which are attributable to the chemical composition of EE, which leads to a slow metabolism with prolonged tissue retention. For this reason, low doses of estrogen constitute an alternative to reduce the thrombotic risk due to low hepatic metabolization. 41

## Contraceptive Use and Hormonal Therapy during the COVID-19 Pandemic

According to recommendations of Spanish societies, women using COCs should immediately suspend the drug and the administration of low molecular weight heparin (LMWH) should be indicated. 35 In addition, according to Paschou et al., 39 for women infected with SARS-CoV-2, any hormone replacement therapy should be suspended until the period of isolation or of hospitalization is over. These positions are based on an apparently logical association between the existing thromboembolic risk with the use of hormones and in severe cases of COVID-19 infection. However, there is no evidence that thromboembolic events in women who use contraceptives have any association or have a worse prognosis with COVID-19. 36

Since thrombosis related to contraceptive use is due to increased clotting factors and hepatic overload, it is not prudent to use a direct association of increased factor in the face of the pathophysiology of the coronavirus to explain a synergism between them. After all, current autopsy reports prove a thrombotic condition of localized origin in the lungs, 25 26 27 just as laboratory predictors are related to an activation of the extrinsic coagulation cascade by endothelial injury and, therefore, do not make the diagnosis for a generalized thrombophilic condition. 2 36

For these reasons and added to the potential protective effect of estrogens in stimulating ACE2, 36 there is no reason to prevent the use of hormones during the COVID-19 pandemic. 7 Therefore, it is recommended to maintain hormonal contraceptive methods as well as to establish special conditions for use during the pandemic. 30 31 32 54 Chart 2 describes the recommendations of the Faculty of Sexual and Reproductive Healthcare (FSRH) for the use of contraception during the COVID-19 pandemic.

**Chart 2:** Recommendations of the Faculty of Sexual and Reproductive Healthcare (FSRH) for the use of contraception during the COVID-19 pandemic

Situation	Recommendation
Request to start contraception	Evaluate remotely – provide desogestrel POPs for 6 to 12 months
Use of medroxyprogesterone acetate (injectable)	Desogestrel POPs
4-year-old etonogestrel implant	No indication of face-to-face consultation for exchange after expiration date – with the exception of women who wish to become pregnant or have serious adverse effects
	Indicated aditional desogestrel POP
Use of 52mg SIU of Levonogestrel	POP for aditional use
5-year-old copper IUD	No indication of face-to-face consultation for exchange after expiration date – with the exception of women who wish to become pregnant, have signs of infection or have serious adverse effects
	Condom use and/or desogestrel POPs
POP is not indicated/appropriate	Complete remote evaluation of eligibility for a COC – AP and BMI accurate and self-referred
	Provide 6- to 12-month supply
	Ineligible patient for COC: injectable medroxyprogesterone, etonogestrel implant and IUD can be considered

Abbreviations: AP, arterial pressure; BMI, body mass index; COC, combined oral contraceptive; IUD, intrauterine device; IUS, intrauterine system; POP, progestogen-only pill.

## Conclusion

Based on female-specific sexual characteristics such as higher expression of ACE2 and TLR7 due the additional chromosome X, as well as on the tissue protection and broad anti-inflammatory action of progesterone and estrogen, it is well-known that women should be analyzed from a particular perspective regarding COVID-19. Therefore, it is established that endogenous hormones have fundamental characteristics that determine a better prognosis for women, as demonstrated in epidemiological studies. However, due to the broad necessity of hormonal treatments for the management of pathologies as well as for contraception, the use of therapies with exogenous hormones, in the context of COVID-19 infection, was put under discussion. Based on current evidence, as well as on the recommendations of the main health agencies of the world, the use of hormonal contraceptives should be maintained during the current pandemic context. Even so, it is highlighted that some formulations with 2 ^nd^ generation progestogens (Levonogestrel), despite having a greater androgenic effect, are the best alternatives for patients with increased risk for thrombosis.
